# Comprehensive analysis of phagocytosis regulatory genes in bladder cancer: implications for prognosis and immunotherapy

**DOI:** 10.3389/fmolb.2025.1608519

**Published:** 2025-06-19

**Authors:** Xueming Ma, Dongnuan Yao, Weitao Yu, Gongping Wu, Chengwei Fan, Junqiang Tian

**Affiliations:** ^1^ Department of Urology, The Second Hospital of Lanzhou University, Lanzhou, China; ^2^ Gansu Province Clinical Research Center for Urinary System Disease, Lanzhou University Second Hospital, Lanzhou, China; ^3^ The Second Hospital and Clinical Medical School, Lanzhou University, Lanzhou, China

**Keywords:** BLCA, phagocytosis regulatory genes, immune infiltration, prognostic, therapeutic response

## Abstract

**Background:**

Bladder cancer is a common malignant tumor of the urinary system. Its incidence and mortality rates are on the rise, and the existing treatment methods are difficult to meet the prognostic needs of patients. Phagocytosis plays a crucial role in tumor immune surveillance and the regulation of the tumor microenvironment. Phagocytosis regulatory genes (PRGs) are involved in regulating the immune response against tumor cells, and in-depth research on them in bladder cancer is extremely urgent.

**Methods:**

Multi-omics data from the TCGA and GEO databases were integrated, and strict data preprocessing was carried out. A variety of algorithms and analysis techniques, such as Kaplan-Meier analysis, Cox regression analysis, and ConsensusClusterPlus clustering analysis, were used to identify PRGs related to the prognosis of bladder cancer patients, and functional analysis and clustering analysis were conducted in depth. A prognostic model was constructed and verified, and the risk score was calculated. At the same time, the relationships between the model and the tumor microenvironment (TME), immune infiltration, mutation, and drug sensitivity were comprehensively analyzed.

**Results:**

It was found that 37 genes had a strong positive correlation with the macrophage score, and 200 PRGs were significantly enriched in immune-related biological processes and pathways. The patients were divided into PRG cluster A and PRG cluster B. Patients in PRG cluster A had a worse survival outcome and were closely related to higher tumor grades, stages, and the infiltration of specific immune cells. A total of 1,696 differentially expressed genes and two phagocytosis-related gene subtypes were identified. The constructed prognostic model showed excellent predictive performance, and the areas under the curves of survival rates at different times were all high in both the training set and the test set. Finally, the drug sensitivity analysis showed that high-risk patients benefited more from immunotherapy and chemotherapy drugs.

**Conclusion:**

This study has greatly deepened the understanding of the potential molecular mechanisms of bladder cancer, provided new insights and valuable potential therapeutic targets for the precision treatment of bladder cancer, and is expected to promote the innovation and optimization of bladder cancer treatment strategies.

## Introduction

Bladder cancer is the second most common malignant tumor of the urinary system globally, with approximately 549,000 new cases and about 200,000 deaths each year (Aaes et al., 2016). Despite the continuous progress of existing treatment methods, the recurrence rate of bladder cancer remains high, and the prognosis of patients is generally poor (Anderson et al., 2021). Therefore, there is an urgent need to develop effective biomarkers to predict prognosis, optimize treatment strategies, and provide a basis for the research and development of new therapies.

Phagocytosis is a core mechanism of the immune system for clearing pathogens, apoptotic cells, and cell debris. The abnormal regulation of phagocytosis is closely related to the remodeling of the tumor immune microenvironment (TME) (Feng et al., 2019). Phagocytosis regulatory genes (PRGs) play a dual role in tumor immune surveillance and escape by regulating the activities of immune cells such as macrophages and dendritic cells (Arlauckas et al., 2021). For example, PRGs can affect the clearance efficiency of tumor cells by regulating processes such as phagosome maturation and antigen presentation (Sun et al., 2021). Abnormal PRG expression correlates with tumor progression, metastasis, and immunotherapy resistance (Madeddu et al., 2022; Sadeghi Rad et al., 2021).

In recent years, the key position of phagocytosis in tumor immunity has been gradually revealed. Studies have shown that the functional heterogeneity of PRGs is closely related to the prognosis of various cancers (Xiao et al., 2024; Feng J. et al., 2022). However, in bladder cancer, systematic research on PRGs is still relatively scarce.

The aim of this study is to systematically analyze the expression characteristics, functional enrichment, and prognostic value of PRGs in bladder cancer. By integrating multi-omics data from the TCGA and GEO databases, combined with consensus clustering, Cox regression, and machine learning algorithms, a prognostic model based on PRGs will be constructed, and its associations with immune infiltration, mutation profiles, and drug sensitivity will be explored. The research results will provide a new theoretical basis for the precision typing, prognostic prediction, and targeted treatment of bladder cancer.

## Materials and methods

### Data collection and processing

mRNA expression data, mutation data, and clinical information of 412 bladder cancer specimens and 19 normal bladder specimens were obtained from the TCGA database (https://portal.gdc.cancer.gov/). To facilitate differential analysis, the fragments per kilobase of exon per million mapped reads (FPKM) values in the TCGA-BLCA cohort were converted into transcripts per million (TPM) values. At the same time, gene expression data and clinical information were obtained from the GSE32894 dataset (*n* = 308) in the GEO database (https://www.ncbi.nlm.nih.gov/geo/).

According to the annotation file, the expression values at the probe level (probe IDs) were accurately converted into the corresponding gene symbols. After strict data quality assessment, it was determined that no further standardization was required. When multiple probes matched the same gene, the expression value of that gene was calculated as the average of the values of these probes. Clinical variables included age, gender, stage, follow-up duration, and survival status.

Before comparing and analyzing the PRG expression, the original data were standardized to the fragment expression level per kilobase. Strict sample exclusion criteria were formulated: patients with a proportion of missing gene expression values exceeding 30% were excluded. Finally, the analysis included 406 BLCA patients from the TCGA dataset and 308 BLCA patients from the GSE32894 dataset.

To eliminate platform-related differences, the “ComBat” method was used to correct the batch effects of the integrated BLCA samples from the TCGA and GEO databases. All data were preprocessed using the “limma” and “sva” R packages (Ritchie et al., 2015).

### Consensus clustering analysis and functional annotation

A total of 247 phagocytosis regulatory genes were identified through a comprehensive review of published literature and the authoritative MsigDB database (https://www.gsea-msigdb.org/gsea/index.jsp). Initially, the survival differences of phagocytosis regulatory genes were carefully analyzed using the Kaplan-Meier (KM) method. The “Limma” software package was used to accurately analyze the expression differences between cancer tissue samples and adjacent normal samples according to the expression profiles of phagocytosis regulatory genes. Univariate Cox regression analysis was performed, and phagocytosis regulatory genes related to prognosis were determined with a strict screening criterion of p < 0.05. Among them, 128 genes showed positive correlation (HR > 1) and 72 showed negative correlation (HR < 1) with overall survival, with median HR of 1.35 (95% CI: 1.21–1.51).

Using the ConsensusClusterPlus R program, a comprehensive identification analysis was carried out for k values from 1 to 9 based on the expression of 200 phagocytosis regulatory genes with survival differences. After repeated verification and evaluation, the optimal number of clusters was determined to be k = 2 (Yao et al., 2024). Based on the mRNA expression of prognosis-related PRGs, principal component analysis (PCA) was used for strict classification verification to ensure the accuracy and reliability of the classification.

To deeply detect the differences in pathway enrichment between PRG clusters, the GSVA algorithm was used to accurately calculate the enrichment score of each gene set, comprehensively exploring the differences in biological functions between PRG clusters. The signature gene sets used in GSVA were all from the authoritative MSigDB database. The “limma” package in R was used to conduct a strict differential analysis between PRG clusters. To ensure the statistical significance of the results, the p-value threshold was strictly set to <0.05 to identify significantly enriched pathways. In addition, the ssGSEA algorithm was used to accurately calculate the infiltration scores of immune cells in each sample according to the characteristic immune cell gene sets, comprehensively evaluating the infiltration levels of various immune cells in tumor samples (Jin et al., 2021). In this study, the ssGSEA algorithm in the “GSVA” R package was used to systematically and comprehensively evaluate the immunological characteristics of each BLCA sample in different PRG clusters.

### Identification and functional analysis of differentially expressed genes (DEGs) related to phagocytosis regulation

The “limma” package in R was used to conduct a strict differential analysis to identify the differentially expressed genes (DEGs) between the two PRG clusters. Strict screening thresholds were set, p < 0.05 and log2 fold change (log2FC) > 0.585, to ensure that the selected differential genes had biological significance (Ritchie et al., 2015). To deeply explore the pathways enriched by the differential genes, the R packages “clusterProfiler” and “org.Hs.eg.db” were used to comprehensively carry out gene ontology enrichment analysis and Kyoto Encyclopedia of Genes and Genomes pathway enrichment analysis, with a critical value of p < 0.05 to ensure the reliability of the analysis results (Kanehisa and Goto, 2000; Ashburner et al., 2000).

### Analysis of phagocytosis regulatory gene subtypes in bladder cancer

For a more in-depth and comprehensive analysis, an unsupervised consensus clustering method was adopted. Using ConsensusClusterPlus R package with 1,000 resampling iterations, 80% sample size, and Euclidean distance metric. Optimal k = 2 was determined by CDF curve and silhouette score (Figure 4D). Subsequently, the gene expression characteristics and clinical significance of these two subtypes were deeply analyzed.

### Construction and verification of the PRGs prognostic model

Based on the identified 1,696 DEGs, univariate Cox regression analysis was used to accurately identify genes related to prognosis with a strict critical value of p < 0.05. To reduce the risk of overfitting, LASSO regression was used for strict feature selection and model optimization. Finally, after repeated verification and evaluation, multivariate Cox regression was used to determine the important genes related to the prognosis of BLCA (Qin et al., 2023; Zhu et al., 2024).

For each patient, the risk score was calculated using the formula: PRG_score = Σ (Expi * coefi) n, where coefi and Expi represent the regression coefficient and expression level of each characteristic gene, respectively. The “caret” R package was used to randomly divide the BLCA samples into a training set and a test set at a ratio of 1:1. Strict balance tests and unbiasedness verifications were carried out to ensure the scientific nature of the grouping (Friedman et al., 2010). Then, according to the median risk score, the samples in the training set and the test set were strictly divided into a high-risk group and a low-risk group. Kaplan-Meier survival analysis and time-dependent receiver operating characteristic (ROC) curves were used to comprehensively evaluate the accuracy of the risk model. The “survival,” “rms,” and “regplot” R packages were used to carefully create a nomogram for predicting the 1-year, 3-year, and 5-year survival rates of patients, and strict verification and calibration were carried out.

### Correlation between prognostic characteristics and TME and immune infiltration

According to the established algorithm process and parameter settings, the ESTIMATE algorithm was executed to accurately estimate the immune cells and stromal cells in BLCA. This algorithm predicts the infiltration levels of immune cells and stromal cells by calculating the immune and stromal scores. To quantify the total number of tumor-infiltrating immune cells in each sample, the CIBERSORT method was applied to strictly compare the infiltration of 21 types of immune cells between the high-risk group and the low-risk group (Chen et al., 2018).

### Mutation and drug sensitivity analysis

Using the “maftools” R package, according to the standard annotation process in the MAF format, the mutation data from the TCGA database were accurately annotated, and the tumor mutation burden (TMB) scores of each bladder cancer patient in the high-risk group and the low-risk group were calculated. Using the R package “pRRophetic,” according to the established prediction model and parameter settings, the half-maximal inhibitory concentration (IC50) of anti-cancer drugs in the high-risk group and the low-risk group was predicted.

### Statistical analysis

All data analyses were performed using R software (version 4.2.2). For the comparison of differences between two groups, an appropriate Wilcoxon rank sum test method was selected according to the data distribution characteristics and research purposes. The Spearman test was used to strictly test the correlation between different variables. The p-value was set to be two-sided, and p < 0.05 was considered to be statistically significant.

## Results

### Correlation between phagocytosis regulators and macrophages

**FIGURE 1:**
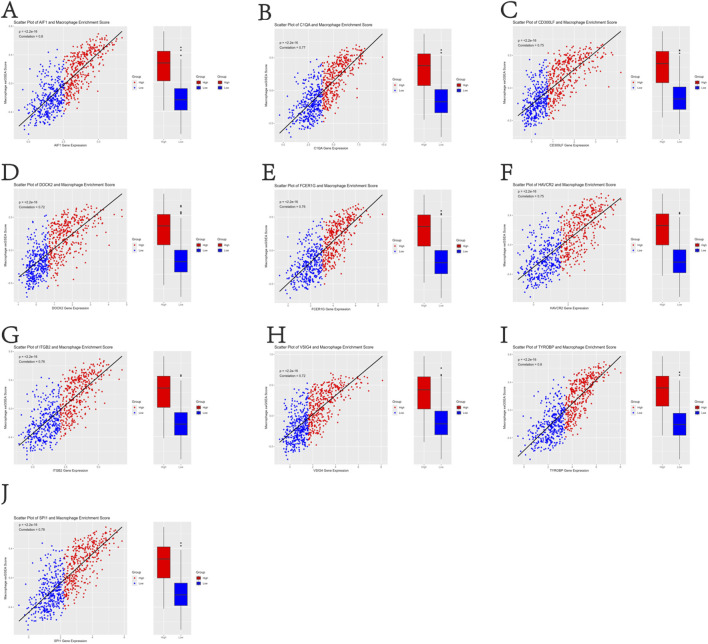
Positive correlation between 37 phagocytosis regulatory genes and macrophage enrichment scores in TCGA-BLCA samples (Pearson’s r > 0.5, p < 0.001) **(A–J)**.

This study focused on the phagocytosis regulators that modulate the phagocytosis of macrophages. Using the characteristic genes of immune cells from the literature PMID28052254, the characteristic genes of macrophages were extracted through a rigorous screening and verification process. Based on the macrophage gene set, the ssGSEA analysis method was used to accurately analyze the enrichment of TCGA-BLCA samples in the macrophage gene set. After obtaining the macrophage enrichment scores, Pearson correlation analysis was used to precisely calculate the correlation between the phagocytic factors and the macrophage enrichment scores. Meanwhile, according to the median expression level of the phagocytic factors, the samples were strictly divided into high-expression and low-expression level groups, and a statistical analysis was conducted on the differences in enrichment scores between the high- and low-expression groups. The results showed that the correlation coefficients of 37 genes were >0.5 and the p-values were <0.001 (Supplementary Table S1). This indicates that many phagocytosis regulators are positively correlated with the macrophage score, and macrophages can significantly distinguish the expression abundance of phagocytosis regulators (Figures 1A–J).

### Functional enrichment analysis of phagocytosis regulators

**FIGURE 2:**
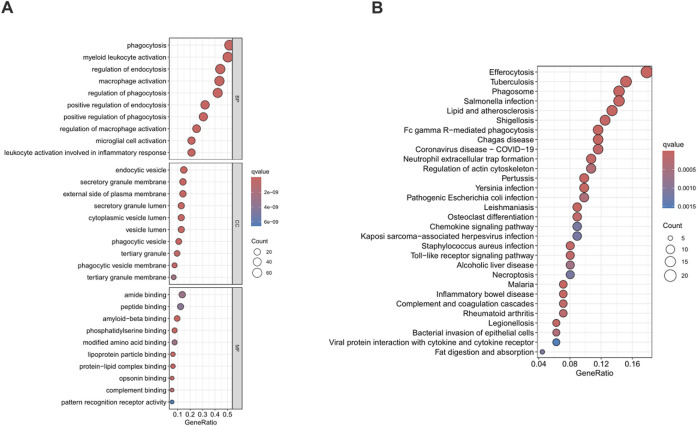
Functional enrichment analysis of phagocytosis regulators. Figure **(A)** shows the results of Gene Ontology (GO), and Figure **(B)** shows the results of Kyoto Encyclopedia of Genes and Genomes (KEGG). GO, Gene Ontology; KEGG, Kyoto Encyclopedia of Genes and Genomes.

A comprehensive functional enrichment analysis was carried out on 200 phagocytosis regulators. With a strict standard of P < 0.05, significantly enriched (148) Gene Ontology (GO) and Kyoto Encyclopedia of Genes and Genomes (KEGG) sets were identified. In the field of biological processes, gene sets related to the phagocytosis and activation of immune cells were significantly enriched, especially in phagocytosis and myeloid leukocyte activation. The analysis of cell components showed significant enrichment of endocytic vesicles and secretory granule membranes. In terms of molecular functions, the binding functions mainly enriched in amide binding and peptide binding were predominant (Figure 2A). The KEGG enrichment analysis showed that phagocytosis regulators were associated with a variety of diseases, especially tuberculosis among infectious diseases and the clearance of apoptotic cells (Figure 2B). The comprehensive analysis shows that the functions of phagocytosis regulators are closely related to the enriched GO categories and KEGG pathways identified in BLCA.

### Expression characteristics and clustering analysis of PRGs

To comprehensively analyze the expression characteristics of PRGs in BLCA, the gene expression matrices from the TCGA and GSE32894 datasets were integrated to construct a comprehensive matrix. Based on the gene expression data of this combined cohort, univariate Cox regression analysis was used to strictly identify 200 phagocytosis regulatory genes (PRGs) that were significantly associated with the overall survival of BLCA patients (Supplementary Table S3).

**FIGURE 3:**
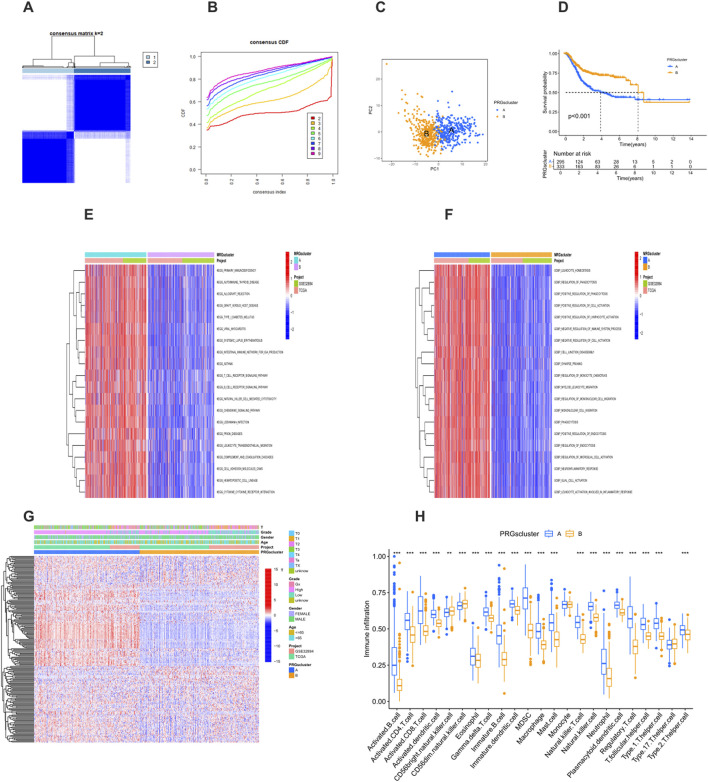
Identification of prognosis-related PRG clusters. **(A)** Consensus clustering heatmap showing two PRG clusters. **(B)** Cumulative distribution function (CDF) curve for consensus clustering. **(C)** Principal component analysis (PCA) showing genomic differences between the two clusters. **(D)** Kaplan-Meier survival curves between PRG Cluster A and Cluster B (P < 0.001). **(E)** Heatmap of Gene Ontology (GO) enrichment analysis between clusters. **(F)** Heatmap of Kyoto Encyclopedia of Genes and Genomes (KEGG) pathway enrichment analysis between clusters. **(G)** Heatmap showing the association of PRG clusters with clinical features and expression levels of prognosis-related PRGs. **(H)** Boxplot comparing immune cell infiltration between PRG Cluster A and Cluster B (P < 0.05). *P < 0.05, **P < 0.01, ***P < 0.001.

By comprehensively testing k values from 1 to 9, a consensus matrix (Figure 3A) and a cumulative distribution function (CDF) curve (Figure 3B) were generated. After repeated verification and evaluation, the optimal number of clusters was determined to be k = 2. This analysis identified two different PRG clusters: PRG cluster A (*n* = 342) and PRG cluster B (*n* = 372) (Supplementary Table S2). The PCA results showed a clear separation between the two clusters (Figure 3C), fully confirming the reliability of the clustering method. Kaplan-Meier survival analysis showed that patients in PRG cluster A had a significantly worse survival outcome compared with those in PRG cluster B (Figure 3D).

### Functional analysis of PRG clusters

The GO analysis showed that PRG cluster A was enriched in functions such as the regulation of leukocyte homeostasis and the regulation of phagocytosis, which play key roles in immune regulation and cell activities (Figure 3E). This cluster was closely related to biological processes such as the positive regulation of cell activation and the positive regulation of lymphocyte activation, highlighting the importance of immune cell activation and the regulation of immune responses. At the same time, in terms of cell components and biological processes, it was involved in processes such as cell junction disassembly and synaptic pruning, reflecting the participation of cell structure and nervous system-related activities. In addition, processes such as the regulation of monocyte chemotaxis and monocyte migration were also reflected in this cluster, further indicating the role of immune cell migration and immune defense mechanisms. The KEGG pathway analysis further showed that cluster A was characterized by the significant enrichment of immune-related pathways, involving multiple aspects such as primary immunodeficiency, autoimmune diseases, transplantation rejection, immune cell signaling, and migration (Figure 3F). This emphasizes the complexity of the body’s immune state and the important therapeutic significance of these immune-related mechanisms in the occurrence and development of diseases. The enrichment of these immune pathways reflects the uniqueness of cluster A at the level of immune regulation and indicates that targeted treatment of these immune-related pathways may be a potential strategy for improving the relevant disease conditions, further highlighting the heterogeneity of diseases and the necessity of precision treatment.

### Relationship between PRG clusters, clinical characteristics, and immune cell infiltration

The heatmap clearly showed the relationship between PRG clusters, PRG expression levels, and clinical characteristics (Figure 3G). Samples in PRG cluster A were associated with higher tumor grades and stages, and the expression levels of prognosis-related PRGs were increased. In addition, there were significant differences in immune cell infiltration between the clusters. Patients in PRG cluster A showed higher infiltration of immune cells, such as activated B cells, activated CD4^+^ T cells, activated CD8^+^ T cells, activated dendritic cells, and myeloid-derived suppressor cells (MDSCs). On the other hand, the levels of CD56 bright natural killer cells and CD56dim natural killer cells were higher in PRG cluster B (Figure 3H). These differences may play a key role in mediating the differences in clinical outcomes.

### Identification and enrichment analysis of DEGs in PRG clusters

**FIGURE 4:**
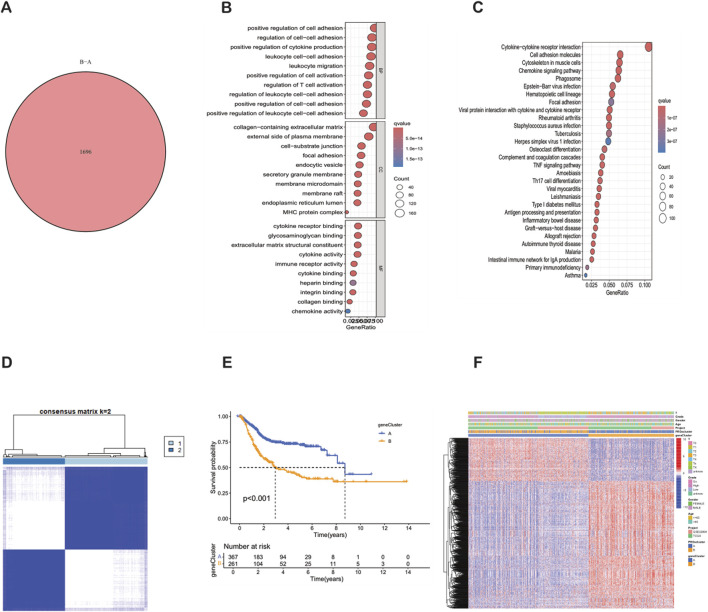
Identification, clustering and functional analysis of differentially expressed genes (DEGs) between PRG clusters. **(A)** Venn diagram showing the 1,696 DEGs identified between PRG clusters. **(B)** Bubble plot of GO enrichment analysis of DEGs. **(C)** KEGG pathway enrichment analysis of DEGs. **(D)** Consensus matrix heatmap defining two gene subtypes (k = 2). **(E)** Kaplan-Meier survival curves between gene Cluster A and Cluster B (P < 0.001). **(F)** Relationships between clinical features and the two gene subtypes.

Using the “limma” R package, according to the strict differential gene identification process, the differentially expressed genes (DEGs) between PRG clusters were identified. The screening criteria were strictly set as |log2FC| > 1 and false discovery rate (FDR) < 0.05. This analysis revealed 1,696 DEGs between PRG cluster A and cluster B (Supplementary Table S4) (Figure 4A). To further elucidate the biological functions of these DEGs, a comprehensive Gene Ontology (GO) and Kyoto Encyclopedia of Genes and Genomes (KEGG) enrichment analysis was performed. The GO analysis showed that the DEGs were mainly involved in the positive regulation of cell adhesion, the regulation of cell adhesion, and leukocyte cell adhesion. The corresponding cell components were mainly located in the collagen-containing extracellular matrix and the outer side of the plasma membrane. In terms of molecular functions, the DEGs were mainly related to cytokine receptor binding and the structural components of the extracellular matrix (Figure 4B). The KEGG analysis showed that the DEGs were significantly enriched in cytokine-cytokine receptor interaction, cell adhesion molecules, and the cytoskeleton in muscle cells (Figure 4C). Further consensus clustering analysis identified two different phagocytosis-related gene subtypes, named subtype A (*n* = 367) and subtype B (*n* = 261), each of which exhibited a unique gene expression profile (Supplementary Table S5) (Figure 4D). Kaplan-Meier survival analysis showed that there w ere significant differences in prognosis between these subtypes, and patients belonging to subtype A had a better outcome (Figure 4E). In addition, it was found that the clinical characteristics of BLCA patients were closely related to these gene sub types (Figure 4F). It is worth noting that significant differences in the expression levels of PRGs were observed in the two phagocytosis factor gene subtypes.

### Construction and verification of the PRGs prognostic model

**FIGURE 5:**
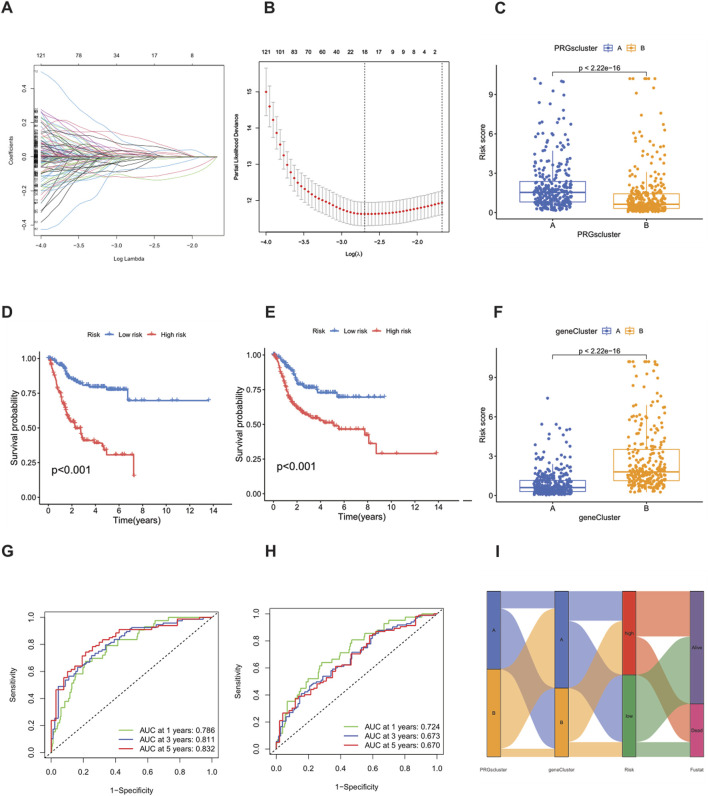
Construction of the prognostic signature based on DEGs between PRG clusters. **(A,B)** LASSO COX regression analysis. **(D,E)** Kaplan-Meier (K–M) curves in the high-risk and low-risk groups of the training set and the test set. **(G)** 1-year, 3-year, and 5-year receiver operating characteristic (ROC) curves in the training set. **(H)** 1-year, 3-year, and 5-year ROC curves in the test set. **(C)** Changes in risk scores between PRG clusters. **(F)** Differences in risk scores between different gene subtypes. **(I)** Sankey diagram showing the correspondence between PRG clusters, gene subtypes, risk scores and survival status.

**FIGURE 6:**
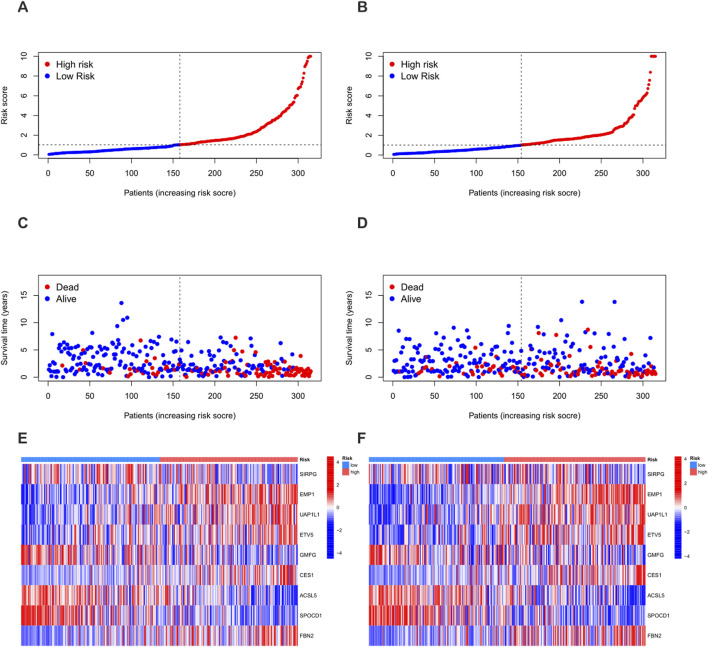
Differences in the distribution of patient survival status and risk scores between the training set and the test set. **(A,C,E)** Distribution of patient survival status and risk scores in the training set. **(B,D,F)** Distribution of patient survival status and risk scores in the test set.

Using the “caret” R package, all patients were randomly divided into a training set and a test set at a ratio of 1:1, and strict balance tests and unbiasedness verifications were carried out. Based on the 1,696 differential genes, univariate Cox regression analysis and Kaplan-Meier survival analysis were first used to accurately identify genes related to prognosis. To improve the gene selection and prevent overfitting, LASSO regression was applied. After multiple feature selections and model optimizations, 18 genes significantly related to prognosis were determined (Supplementary Table S6) (Figures 5A,B). Subsequently, multivariate Cox regression analysis reduced the list to nine key genes—SIRPG, EMP1, UAP1L1, ETV5, GMFG, CES1, ACSL5, SPOCD1, and FBN2—for constructing the prognostic model (Supplementary Table S7). The risk score of each patient was calculated as follows: Risk score = (−0.2879 × SIRPG expression) + (0.3339 × EMP1 expression) + (0.3076 × UAP1L1 expression) + (0.2508 × ETV5 expression) + (−0.2017 × GMFG expression) + (0.1174 × CES1 expression) + (−0.1562 × ACSL5 expression) + (−0.1659 × SPOCD1 expression) + (0.0965 × FBN2 expression). According to the median risk score, the patients in the training set and the test set were strictly divided into a high-risk group and a low-risk group (Figures 6A–F). Kaplan-Meier survival analysis showed that, in both cohorts, the overall survival (OS) of high-risk BLCA patients was significantly worse compared with that of low-risk patients (Figures 5D,E). The prognostic performance of the model was further evaluated using ROC analysis, and the areas under the curves (AUCs) of the 1-year, 3-year, and 5-year survival rates in the training set were 0.786, 0.811, and 0.832, respectively (Figure 5G). In the test set, the AUC values of the 1-year, 3-year, and 5-year survival rates were 0.724, 0.673, and 0.670, respectively (Figure 5H), strongly demonstrating the powerful predictive accuracy of the gene signature for the prognosis of BLCA. In addition, significant differences in risk scores were observed between the PRG clusters, and the risk score of PRG cluster A was higher than that of PRG cluster B (Figure 5C). In the phagocytosis factor-related gene clusters, significant differences in risk scores were determined, and patients in the gene cluster subtype A had a better outcome (Figure 5F). The Sankey diagram further showed the distribution of patients in the two PRG score groups, the two phagocytosis factor-related clusters, and the two gene subtypes, revealing that most patients in PRG cluster B were related to gene subtype B, which had a lower risk score and a correspondingly better prognosis (Figure 5I).

### Construction of the prognostic nomogram

**FIGURE 7:**
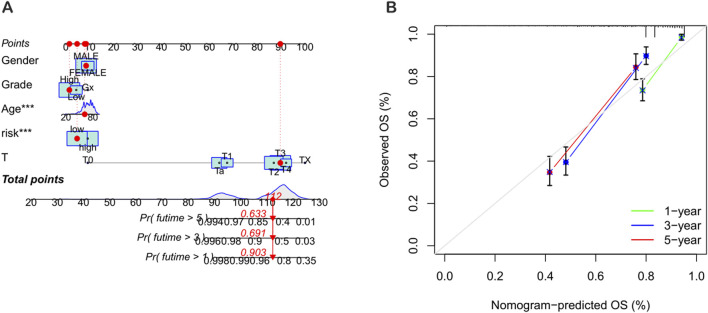
Construction and validation of the nomogram. **(A)** Nomogram for predicting the 1-year, 3-year, and 5-year overall survival (OS) rates of BLCA patients. **(B)** Calibration curve of the OS nomogram model.

To improve the accuracy of predicting the prognostic outcomes of bladder cancer patients, a nomogram was carefully constructed, which integrated the patient’s age, pathological stage, and the risk score derived from the prognostic model. This nomogram provides a comprehensive tool for estimating the 1-year, 3-year, and 5-year overall survival (OS) probabilities of patients (Figure 7A). The red mark in the nomogram shows an example prediction, indicating that the higher the total score, the worse the prognosis. The calibration plot confirmed the predictive reliability of the nomogram, showing a strong consistency between the predicted survival rate and the observed survival rate (Figure 7B).

### Relationship between the tumor microenvironment (TME), immune infiltration, and PRG score

**FIGURE 8:**
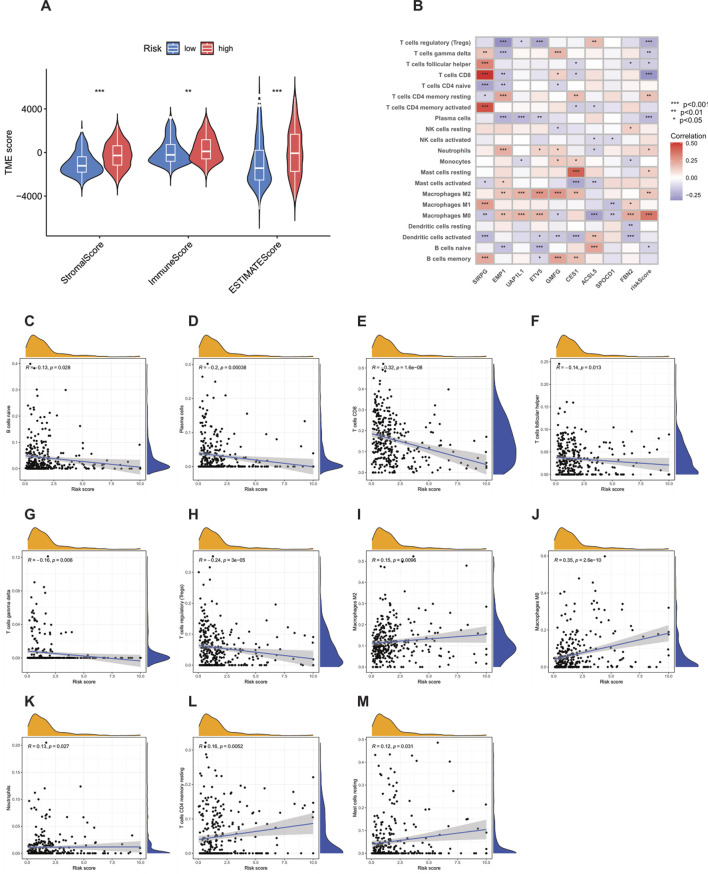
Analysis of the correlation between the tumor microenvironment (TME) and immune cell types in the high-risk and low-risk groups. **(A)** Differences in ImmuneScore, StromalScore, and ESTIMATEScore between the high-risk and low-risk groups. **(B–M)** Correlation between the risk score and immune cells.

Using the ESTIMATE algorithm, according to the established algorithm process and parameter settings, the differences in TME scores between the high-risk group and the low-risk group were first calculated (Supplementary Table S8). The analysis results showed that compared with the high-risk group, the stromal score (StromalScore), immune score (ImmuneScore), and ESTIMATE score of the low-risk group were significantly lower (Figure 8A). In addition, using the CIBERSORT algorithm, according to the standardized process, the proportions of tumor-infiltrating immune cells in each TCGA-BLCA sample were calculated (Supplementary Table S9). Subsequently, Spearman correlation analysis was performed to rigorously explore the association between the PRG-based prognostic score (PRG_score) and immune cell infiltration. The results showed that naive B cells (R = −0.13, p = 0.028, Figure 8C), plasma cells (R = −0.2, p = 0.00038, Figure 8D), CD8 T cells (R = −0.32, p = 1.6e−08, Figure 8E), follicular helper T cells (R = −0.14, p = 0.013, Figure 8F), γδ T cells (R = −0.16, p = 0.006, Figure 8G), and regulatory T cells (Tregs, R = −0.24, p = 3e−05, Figure 8H) were negatively correlated with the risk score, while M2 macrophages (R = 0.15, p = 0.0096, Figure 8I), M0 macrophages (R = 0.35, p = 2.6e−10, Figure 8J), neutrophils (R = 0.13, p = 0.027, Figure 8K), resting memory CD4 T cells (R = 0.16, p = 0.0052, Figure 8L), and resting mast cells (R = 0.12, p = 0.031, Figure 8M) were positively correlated with the risk score.

The strong positive correlation between M0 macrophages and the high-risk score is particularly worthy of in-depth exploration. As unpolarized macrophages, M0 macrophages have the potential to differentiate into M1-type (pro-inflammatory, anti-tumor) or M2-type (anti-inflammatory, pro-tumor) macrophages. In this study, the infiltration of M0 macrophages in high-risk patients was significantly increased, which may imply that the tumor microenvironment (TME) has a key impact on the polarization of macrophages, leading to a large number of M0 macrophages existing in the tumor tissue and possibly tending to differentiate into M2-type macrophages, thus creating an immunosuppressive TME. In view of this, targeted regulation of the polarization of M0 macrophages may become a very promising therapeutic approach to improve the prognosis of high-risk cancer patients.

In addition, an in-depth analysis of the relationship between the nine key genes used to construct the prognostic model and various immune cells revealed significant correlations between most immune cell types and these genes (Figure 8B). For example, the expression of FBN2 was positively correlated with M0 macrophages, indicating its potential role in regulating the immune response in the tumor microenvironment. The results further revealed the importance of these genes as potential therapeutic targets.

### Mutation analysis

**FIGURE 9:**
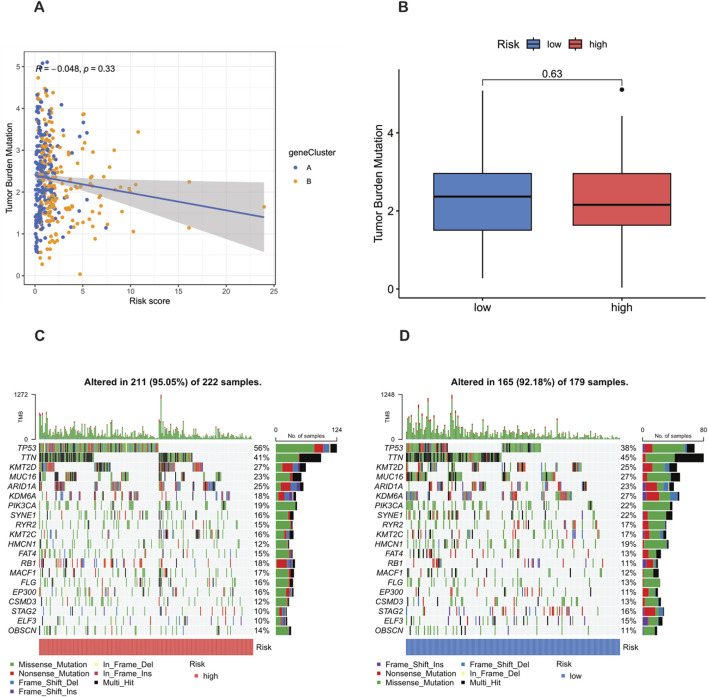
Analysis of tumor mutation burden (TMB) and mutations in high-risk and low-risk populations. **(A)** TMB in the high-risk and low-risk groups. **(B)** Relationship between NRG_score and TMB. **(C,D)** Waterfall plots characterizing somatic mutations determined by high and low NRG scores.

Previous studies have shown that the tumor mutation burden (TMB) can be used as a predictive biomarker for immunotherapy, and generally, a higher TMB is associated with a better response to immunotherapy. However, the analysis of this study showed that there was no significant difference in the TMB scores between the high-risk group and the low-risk group (p = 0.63) (Figure 9B), indicating that the two groups may have a limited response to immunotherapy. In addition, Spearman correlation analysis showed that there was no significant association between the TMB and the risk score in either the high-risk group or the low-risk group (R = −0.048, p = 0.33) (Figure 9A). This finding implies that the prognostic differences between these groups are not driven by changes in TMB, but may be driven by other molecular or microenvironmental factors.

In terms of tumor somatic mutations, this study rigorously observed that the overall mutation rate in the high-risk group (95.05%) was higher than that in the low-risk group (92.18%), and TP53 was the most frequently mutated gene in both groups. Specifically, the mutation frequency of TP53 reached 56% in the high-risk group and 38% in the low-risk group, indicating its key role in the tumor progression and invasiveness of bladder cancer. Other major mutated genes shared between the two groups included TTN, KMT2D, MUC16, ARID1A, KDM6A, PIK3CA, SYNE1, RYR2, and KMT2C (Figures 9C,D). The higher mutation frequency of key oncogenes in the high-risk population indicates increased genetic instability, which may lead to worse clinical outcomes.

### Drug sensitivity analysis

**FIGURE 10:**
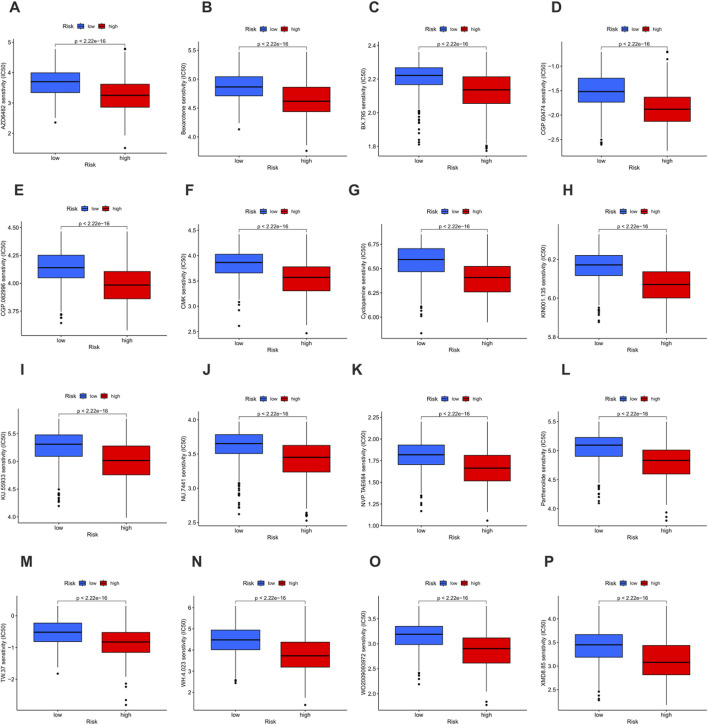
Drug sensitivity analysis based on risk groups. **(A–O)** Boxplots showing the half maximal inhibitory concentration (IC50) values of 16 therapeutic agents in the low-risk group (blue) and the high-risk group (red). Lower IC50 values indicate higher drug sensitivity. **(A)** AZD6482. **(B)** Bexarotene. **(C)** BX - 795. **(D)** CGP - 60474. **(E)** CGP - 082996. **(F)** CMK. **(G)** Cyclopamine. **(H)** KIN001 - 135. **(I)** KU - 55933. **(J)** NU - 7441. **(K)** NVP - TAE684. **(L)** Parthenolide. **(M)** TW - 37. **(N)** WH - 4 - 023. **(O)** WO2009093972. **(P)** XMD8 - 85.

Drug sensitivity was evaluated according to the half maximal inhibitory concentration (IC50) value, and a lower IC50 value indicates greater sensitivity to the treatment. In this study, a total of 16 drugs were strictly screened and determined to be more effective for the high-risk group, including AZD6482, Bexarotene, BX - 795, CGP - 60474, CGP - 082996, CMK, Cyclopamine, KIN001 - 135, KU - 55933, NU - 7441, NVP - TAE684, Parthenolide, TW - 37, WH - 4 - 023, WO2009093972, and XMD8 - 85 (Figures 10A–P). These findings highlight a group of therapeutic drugs that show greater efficacy in the high-risk population, providing valuable insights into the potential drug selection for personalized treatment strategies.

## Discussion

Phagocytosis, a fundamental mechanism of the immune system, plays a crucial role in eliminating pathogens, apoptotic cells, and cellular debris (Feng et al., 2019). Dysregulation of this process is intricately linked to the remodeling of the tumor microenvironment (TME), and phagocytosis regulatory genes (PRGs) have been recognized to exert a dual influence on tumor immune surveillance and escape by modulating the activities of immune cells such as macrophages and dendritic cells (Arlauckas et al., 2021). However, the comprehensive understanding of PRGs in bladder cancer (BLCA) remains limited, and this study aimed to fill this gap through a systematic investigation.

In this study, we conducted an in-depth analysis of PRGs in BLCA by integrating data from TCGA and GEO databases. Our findings revealed a significant positive correlation between many phagocytosis regulators and macrophage scores. Specifically, 37 genes demonstrated a correlation coefficient greater than 0.5 and a p-value less than 0.001, indicating that macrophages can distinctly differentiate the expression abundance of phagocytosis regulators. This correlation provides a novel perspective for deciphering the regulatory mechanisms of the immune microenvironment in BLCA, highlighting the pivotal role of the interaction between macrophages and phagocytosis regulators in tumor immune processes (Weiskopf and Weissman, 2015; Hirayama et al., 2017; Hu et al., 2019).

Functional enrichment analysis of 200 phagocytosis regulators demonstrated their significant enrichment in immune-related biological processes and pathways. Genes related to immune cell phagocytosis and activation, particularly in phagocytosis and myeloid leukocyte activation, were notably enriched in the biological process domain. These findings suggest that phagocytosis regulators are closely associated with the occurrence, development, and immune escape of BLCA, potentially influencing tumor cell clearance and immune cell activation states (Cheng et al., 2024; Xiao et al., 2023).

The expression characteristics and clustering analysis of PRGs unveiled the molecular heterogeneity of BLCA. We identified two distinct PRG clusters (PRG cluster A and PRG cluster B) with significant differences in survival outcomes, as confirmed by Kaplan-Meier analysis. PRG cluster A was associated with a poorer survival prognosis compared to cluster B. Additionally, GO analysis indicated that PRG cluster A was enriched in functions related to leukocyte homeostasis regulation and phagocytosis regulation, highlighting its involvement in immune regulation and cellular activities. KEGG pathway analysis further emphasized the significant enrichment of immune-related pathways in cluster A, suggesting potential therapeutic strategies targeting these pathways to improve disease conditions.

The relationship analysis among PRG clusters, clinical characteristics, and immune cell infiltration showed that samples in PRG cluster A were associated with higher tumor grades and stages, as well as elevated expression levels of prognosis-related PRGs. Moreover, there were significant differences in immune cell infiltration between the clusters. Patients in PRG cluster A exhibited higher infiltration of activated immune cells, such as activated B cells, activated CD4^+^ T cells, and activated CD8^+^ T cells, while PRG cluster B had higher levels of CD56 bright and CD56dim natural killer cells. These differences may play a crucial role in mediating the variation in clinical outcomes.

We identified 1,696 differentially expressed genes (DEGs) between PRG clusters and conducted GO and KEGG enrichment analyses. The DEGs were mainly involved in the positive regulation of cell adhesion, regulation of cell adhesion, and leukocyte cell adhesion. Consensus clustering analysis further defined two distinct Phagocytosis Regulatory Genes subtypes with significant differences in prognosis and clinical characteristics, and notable disparities in PRG expression levels were observed between the subtypes. These findings contribute to a deeper understanding of the molecular mechanisms underlying BLCA and may guide personalized treatment strategies.

A prognostic model for programmed cell death-related genes (PRGs) was constructed and validated using a series of regression analyses and machine learning algorithms. Nine key genes (SIRPG, EMP1, UAP1L1, ETV5, GMFG, CES1, ACSL5, SPOCD1, and FBN2) were identified for predicting the prognosis of bladder cancer patients. As oncogenes, EMP1, UAP1L1, ETV5, CES1, and FBN2 promote tumor deterioration through immune escape, invasion and metastasis, and chemotherapy resistance (Wang Q. et al., 2025; Wu et al., 2022; di Martino et al., 2019; Wang JF. et al., 2025; Lu et al., 2023).

In contrast, as tumor suppressor genes, SIRPG, GMFG, ACSL5, and SPOCD1 inhibit tumor development through metabolic regulation, transcriptional repression, and cell adhesion (Çoban et al., 2023; Gaisa et al., 2013). The risk score calculated based on these genes could effectively stratify patients into high-risk and low-risk groups, with high-risk patients having a significantly poorer overall survival. The prognostic performance of the model, as evaluated by ROC analysis, demonstrated strong predictive accuracy, with AUC values for 1-year, 3-year, and 5-year survival rates in both the training and testing sets indicating its reliability. Additionally, the nomogram constructed by integrating patient age, pathological stage, and risk score provided a comprehensive tool for estimating the survival probability of patients, and the calibration plot confirmed its predictive reliability.

Analysis of the relationship between the tumor microenvironment (TME), immune infiltration, and PRG scores showed that the low-risk group had significantly lower stromal scores, immune scores, and ESTIMATE scores compared to the high-risk group. Spearman correlation analysis revealed significant correlations between various immune cells and the risk score. Notably, the strong positive correlation between M0 macrophages and the high-risk score suggests that the TME may influence macrophage polarization, potentially promoting the differentiation of M0 macrophages into M2 macrophages and creating an immunosuppressive TME. Targeting the polarization of M0 macrophages may represent a promising therapeutic approach for improving the prognosis of high-risk cancer patients (Feng H. et al., 2022).

Regarding mutation analysis, our study found that there was no significant difference in tumor mutation burden (TMB) scores between the high-risk and low-risk groups, and no significant correlation between TMB and the risk score. However, the overall mutation rate was higher in the high-risk group, with TP53 being the most frequently mutated gene in both groups. The higher mutation frequency of key oncogenes in the high-risk group indicates increased genetic instability, which may contribute to poorer clinical outcomes (Li et al., 2023).

In terms of drug sensitivity analysis, we identified 16 drugs that were more effective for the high-risk group based on IC50 values. These findings provide valuable insights for potential drug selection in personalized treatment strategies for BLCA patients.

Undoubtedly, our research based on public databases still has certain limitations. The sample size is limited, and the prognostic model requires further *in vitro* experimental studies and clinical trials to verify its accuracy.

## Conclusion

In conclusion, this comprehensive analysis of PRGs in BLCA has provided important theoretical insights and potential directions for a better understanding of the pathogenesis, prognostic evaluation, and development of therapeutic targets for BLCA. Our findings highlight the significance of PRGs in the molecular heterogeneity, immune regulation, and prognosis of BLCA, and suggest potential therapeutic strategies targeting the interaction between PRGs and the TME. Further research is warranted to translate these findings into clinical practice and improve the outcomes of BLCA patients.

## Data Availability

The datasets presented in this study can be found in online repositories. The names of the repository/repositories and accession number(s) can be found in the article/Supplementary Material.
